# An insect trap adjusting to weather conditions: *Nepenthes rafflesiana* plants control the fluid level in their pitchers to maximize prey capture

**DOI:** 10.1093/aob/mcaf294

**Published:** 2025-11-12

**Authors:** C N S Andrew, J Y Bu, N S Kelly, S Johnson, F Metali, T U Grafe, U Bauer, W Federle

**Affiliations:** Department of Zoology, University of Cambridge, Cambridge CB2 3EJ, UK; Department of Zoology, University of Cambridge, Cambridge CB2 3EJ, UK; Department of Mechanical Engineering, University of Bath, Bath BA2 7A, UK; Department of Biosciences, University of Exeter, Exeter EX4 4QD, UK; Faculty of Science, Universiti Brunei Darussalam, Bandar Seri Begawan, Gadong BE1410, Brunei Darussalam; Faculty of Science, Universiti Brunei Darussalam, Bandar Seri Begawan, Gadong BE1410, Brunei Darussalam; Department of Biosciences, University of Exeter, Exeter EX4 4QD, UK; Department of Zoology, University of Cambridge, Cambridge CB2 3EJ, UK

**Keywords:** Carnivorous plants, plant–insect interactions, ants, plant physiology, digestive fluid, insect capture, digestive glands

## Abstract

**Background and Aims:**

Many carnivorous plants rely on open fluid pools or droplets to trap and digest arthropod prey. Here, we investigate, for *Nepenthes rafflesiana* pitcher plants, how they maintain functional fluid pools inside their traps while growing in open sites exposed to changing weather conditions. We hypothesized that very low or high fluid levels reduce the trapping success of the pitcher and that pitchers possess mechanisms to minimize fluctuations of the fluid level.

**Methods:**

Natural fluid levels of *N. rafflesiana* pitchers were monitored in the field. Effects of the fluid level on prey capture rate and efficiency were quantified with field and laboratory experiments. To test the capacity of plants to respond to changes in fluid level, we experimentally simulated flooding by adding water to pitchers and simulated evaporation by replacing the contents with a smaller volume of concentrated pitcher fluid.

**Key Results:**

Freshly opened *N. rafflesiana* pitchers were approximately half-filled with fluid. Over a 5-week observation period, daily fluctuations of pitcher fluid levels were significantly lower than those of water-filled control vials. Pitchers possess canopy-like lids, but this did not eliminate rainwater influx into pitchers. Both low and very high fluid levels were detrimental to prey capture, with intermediate fluid levels yielding the highest trapping rate. Experimentally flooded pitchers returned to intermediate fluid levels within 2–3 days. Pitchers responded to simulated evaporation by secreting fluid, restoring intermediate fluid levels within 2 days. These homeostatic responses might be triggered by fluid volume or by water potential gradients resulting from changes in concentration.

**Conclusions:**

*Nepenthes rafflesiana* pitchers regulate their fluid level and remain efficient insect traps in fluctuating weather conditions. This active control is a previously unrecognized pitcher plant adaptation to their exposed habitats; understanding it is important for predicting the ability of these plants to withstand extreme weather conditions enhanced by climate change.

## INTRODUCTION

Plant carnivory evolved convergently in ≥12 plant families as a strategy to thrive on nutrient-poor soils and evade competition from other plant species (Fleischmann *et al.*, 2018). All carnivorous plants rely at least temporarily on accumulated or secreted liquids for the release and uptake of nutrients from captured prey. Most species also rely on openly accessible fluid droplets or pools for prey capture and retention. Examples are the flypaper traps of *Drosera*, *Drosophyllum*, *Triphyophyllum*, *Roridula*, *Byblis*, *Pinguicula* and *Philcoxia*, and the pitfall traps of bromeliads (*Brocchinia* and *Catopsis*) and pitcher plants (*Sarracenia*, *Darlingtonia*, *Heliamphora*, *Cephalotus* and *Nepenthes*). The presence of open fluid pools in carnivorous plants, and their resulting dependence on an abundant water supply (Osunkoya *et al.*, 2007), raises the question of how the plants can maintain these functionally important fluid pools in both wet and dry weather conditions (Kingsolver, 1979, 1981). Carnivorous plants typically grow in relatively open habitats, such as bogs or heath forests (Clarke, 1997; Latiff *et al.*, 2014), exposing them to flooding or desiccation (Kingsolver, 1981; Clarke, 1997; McPherson, 2023). Here, we investigate the effect of weather fluctuations on exposed fluids, using tropical *Nepenthes rafflesiana* Jack pitcher plants as a model.

Pitcher plants possess modified funnel-shaped leaves that attract, capture and digest prey (Ellison and Adamec, 2018; Gilbert *et al.*, 2025). Insects are attracted by nectar, olfactory and visual signals (Bennett and Ellison, 2009; Di Giusto *et al.*, 2010; Moran and Clarke, 2010). Anti-adhesive surfaces lining the pitcher rim (peristome) (Bohn and Federle, 2004) and inner wall (Gaume *et al.*, 2002, 2004) cause insects to slip and fall into a pool of digestive fluid contained within the pitcher, where they drown.

The pitcher fluid in some species has viscoelastic properties (Bonhomme *et al.*, 2011; Bazile *et al.*, 2015; Collett *et al.*, 2015), which increases the efficiency of the fluid to retain prey (Gaume and Forterre, 2007; Kang *et al.*, 2021). It has been shown that even in diluted form, the sticky fluid is still very effective at retaining prey (Gaume and Forterre, 2007). In contrast to dilution, the influence of pitcher fluid volume on prey capture in *Nepenthes* has yet to be investigated.

**Fig. 1.**
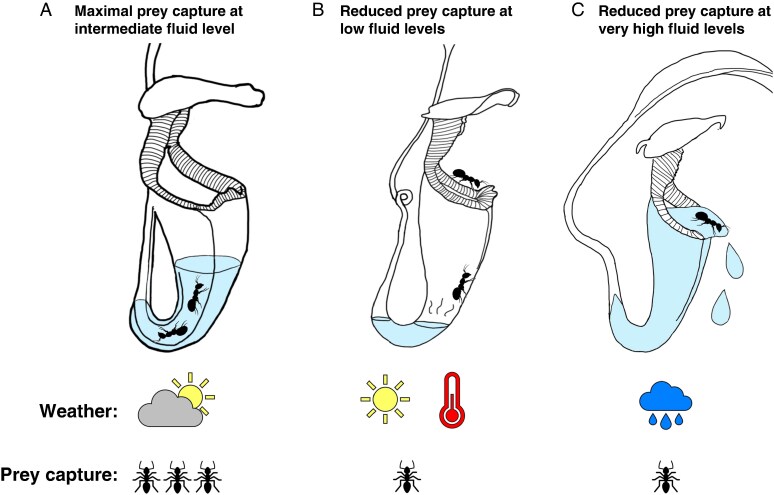
Hypothesized effect of pitcher fluid level on prey capture in *Nepenthes rafflesiana* pitchers. (A) At intermediate fluid levels, prey may fall into the pitcher fluid and cannot escape. (B) At lower fluid levels, prey falling from the peristome may not land directly in the fluid and may be able to climb to safety. (C) At very high fluid levels, prey capture may be less likely, and previously caught prey may be spilled.

Here, we not only study how fluid level impacts the success of prey capture in the natural habitat, but we also investigate the ability of *N. rafflesiana* to respond to rainwater ingress or evaporative fluid loss from its traps. Despite the presence of a canopy-like lid partly sheltering the trap (Lloyd, 1942), pitchers can become flooded during heavy rain or their fluid levels could decrease in dry conditions. Both extremes could have negative consequences for prey capture: when the fluid level is very high, captured prey could escape more easily and be spilled out with the liquid, whereas when the fluid level is very low, insects might not land in the fluid but on the wall of the conical pitcher, making it easier for them to escape (Fig. 1). If the fluid level does affect the capture success, pitcher plants would benefit from the ability to regulate their liquid volume in order to compensate for changing weather conditions.

Previous studies in *N. rafflesiana* showed that the trapping rate increases during the first 2 weeks after opening, then declines in pitchers approaching the end of their 6- to 10-week life span (Di Giusto *et al.*, 2008; Osunkoya *et al.*, 2008; Bauer *et al.*, 2009; Bazile *et al.*, 2015). If pitchers regulate their fluid level, this ability might decrease with age, in line with capture rate.

We systematically investigated the implications of the fluid level on prey capture and the regulatory capacity of plants in a series of experiments in the natural habitat of *N. rafflesiana* in Brunei, Northern Borneo, by addressing the following hypotheses:

1. Pitcher fluid levels fluctuate owing to rainwater ingress and evaporation, despite the shielding by the pitcher lid [Experiment (Exp.) 1].
2. The pitcher fluid level impacts the trapping success of the pitcher. Very low and very high fluid levels reduce prey capture rate and efficiency (Exp. 2).
3. Pitchers actively maintain intermediate fluid levels by replenishing removed fluid or absorbing added water (Exp. 3).
4. The ability to control the fluid level depends on pitcher age (Exp. 4).
5. On the inner pitcher wall, the glands enabling fluid level control differ morphologically between the upper and lower parts of the pitcher.

## MATERIALS AND METHODS

All experiments were performed on *Nepenthes rafflesiana* in its natural habitat at a ∼2 ha site of secondary heath forest near Tutong, Brunei, Borneo (4°44′23.9″N, 114°35′36.4″E) during two field trips (January–June 2023 and June–October 2024). *Nepenthes* pitcher plants typically produce two morphologically distinct trap types: (1) ‘lower’ pitchers, which are ovoid in shape and mostly stand on the ground; and (2) ‘upper’ pitchers, which are funnel-shaped and grow on mature climbing vines, usually suspended above ground level. Field experiments were conducted only on upper pitchers owing to their higher abundance and their more exposed positions in comparison to lower pitchers, which are often sheltered by foliage or even partly submerged in puddles. We conducted different pitcher treatments that were performed in parallel for each experiment.

We measured the natural fluctuations of the pitcher fluid level (Exp. 1), studied the effect of pitcher fluid level on prey capture rate and efficiency (Exp. 2), quantified the response of the pitcher to flooding and fluid evaporation (Exp. 3), and tested the age dependence of the ability of the pitcher to absorb and secrete fluid (Exp. 4).

### Experiment 1: natural fluctuations of pitcher fluid level

To establish the range of naturally occurring fluid levels found in *N. rafflesiana* pitchers and to investigate the extent to which weather conditions cause pitcher fluid levels to fluctuate, we recorded pitcher fluid levels alongside the natural rainfall and examined the effect of the pitcher lid on the fluid level fluctuations. Rainfall at the field site was monitored in intervals of 5 min using a rain gauge (Rain Collector II 7395-024, Davis Instruments, CA, USA; resolution: 0.22 mm per count) connected to a datalogger (TGP-4901, Gemini Data Loggers, Chichester, UK).

**Fig. 2.**
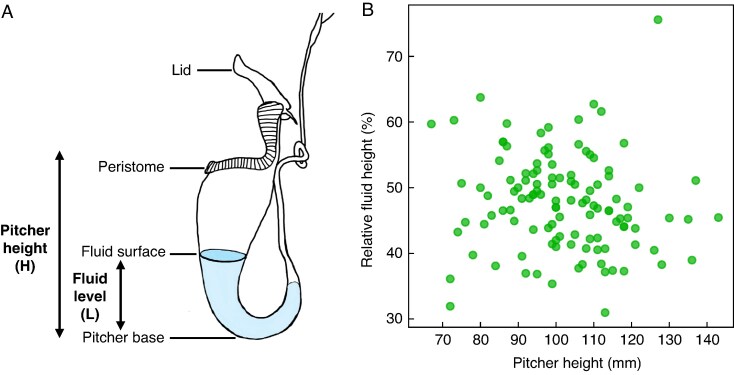
Measurement of the fluid height in *Nepenthes rafflesiana* pitchers. (A) Diagram showing the definition of fluid level *L* (pitcher base to fluid surface) and pitcher height *H* (pitcher base to peristome). (B) Relative fluid height (*L*/*H*) observed in freshly opened *N. rafflesiana* pitchers against pitcher height. We did not find any significant dependence of relative fluid height on pitcher height (*t*_114_ = −1.25, *P* = 0.21).

Pitchers were tagged upon the day of lid opening, which marked day 0 of the experiments. Pitcher fluid level measurements were taken by backlighting the pitcher with a torch and marking the fluid level on the outside of the pitcher with a marker pen. The fluid level was recorded as the distance from pitcher base to the level mark, measured with callipers (*L*, see Fig. 2A). At the end of the pitcher fluid level measurements, fluid volumes were determined from the recorded fluid levels by pipetting water in 0.1 mL increments up to each level mark on the pitcher. Pitcher fluid level measurements were taken for the following experiments.

*Experiment 1a: fluid level in freshly opened and 4-week-old pitchers.* We recorded the fluid level (*L*) on the day of opening in 116 pitchers on separate plants (growing ≥5 m apart) alongside the pitcher height (*H*, measured from pitcher base to peristome, see Fig. 2A) to calculate the average relative pitcher fluid level (*L*/*H*). We also recorded fluid levels in a separate dataset of 12 randomly selected 4-week-old pitchers from different plants.

*Experiment 1b: daily changes of pitcher fluid level.* To measure the changes in pitcher fluid level over time, we recorded the initial fluid level in ten fresh (day 0) pitchers, each on a separate plant. An open Falcon tube of 30 mm diameter (similar to that of a pitcher), prefilled with 10 mL of distilled water, was attached adjacent to each pitcher to differentiate between pitcher fluid level regulation and weather-related fluid level changes. We measured the fluid levels of pitchers and their corresponding Falcon tubes daily at 09.00 h for a period of 35 days (dataset A, five pitchers) and 11 days (dataset B, five pitchers; this second time series was cut short by the destruction of the experimental plants). Daily changes in pitcher or vial fluid levels were calculated by subtracting the fluid level from the previous day (*L_n_* − *L_n_*_−1_).

*Experiment 1c: effect of pitcher lid on pitcher fluid level.* To investigate the effect of the pitcher lid on fluid level fluctuations, we selected 41 open pitchers (<1 week after opening). The lids of ten pitchers were cut off with a scalpel at the junction between the lid and the peristome, and 31 pitchers were left with their lids attached. We recorded pitcher fluid levels daily at 09.00 h for a period of 13 days; daily fluid level changes were calculated as above.

### Experiment 2: influence of pitcher fluid level on prey capture rate and efficiency

To investigate the effect of pitcher fluid level on prey capture, we compared pitchers with different fluid levels by measuring: (1) the prey capture rate in the field; and (2) the prey capture efficiency in a laboratory-based experiment with ants.

*Experiment 2a: field measurement of prey capture rate.* We selected 36 freshly opened pitchers from separate plants and recorded the initial pitcher fluid level. The fluid level was reduced by discarding 50 % of the fluid (low, *n* = 10) or increased by adding 50 % more pitcher fluid (high, *n* = 10) or maximized by filling the pitcher up to the level of the peristome (full, *n* = 6) or left unchanged [intermediate (control), *n* = 10]. Fluid added to pitchers was taken from other freshly opened pitchers that were not part of the experimental treatment. The experimental treatments created a range of fluid heights from 22.0 to 93.6 % of the total pitcher height (measured from peristome to pitcher base). We inserted a conical polyurethane ear plug (Pura-Fit Moldex 7700, Moldex-Metric, Walddorfhäslach, Germany) into the tapered bottom end of each pitcher to prevent prey items getting into this area and becoming inaccessible. We collected prey daily at 07.00 h using a 50 mL syringe to remove the fluid from the pitcher into a Petri dish, where prey items were counted. Prey was then stored in an ethanol-filled tube and later sorted into flying and non-flying species. The pitcher fluid, free from prey, was poured back into the pitcher, and the fluid level was returned to the set height if it had changed slightly in the previous 24 h. We continued sampling for a period of 28 days, after which prey capture began to taper off (Bauer *et al.*, 2009).

*Experiment 2b: laboratory measurements of prey capture efficiency.* To measure trapping efficiency, a colony of *Polyrhachis triaena* ants (a common prey of *N. rafflesiana*) was collected at the study site. The ants were kept in a container with food provided *ad libitum* for 24 h prior to the experiment. A *N. rafflesiana* pitcher was ablated from its parent plant and placed into the container with the ant colony. The peristome was kept wet during the experiment to maintain its slipperiness. The pitcher fluid level was set sequentially to either 25, 50, 75 or 95 % of the pitcher base to peristome height. Ants were allowed to run freely over the pitcher during each experiment. For each fluid level, 20 ants that had slipped from the peristome and fallen into the pitcher were monitored for 10 min. They were recorded as either ‘captured’ (still inside the pitcher after 10 min) or ‘escaped’ (outside the pitcher after leaving it via the peristome). After completing the observations for one fluid, all ants were removed from the pitcher, and the fluid was set to the next level.

### Experiment 3: effect of experimental flooding and fluid evaporation on pitcher fluid levels

To test the response of the pitcher to naturally occurring weather conditions, we manipulated the pitcher fluid in the field to simulate the evaporative effects of drought and diluting effects of heavy rainfall. Flooding was simulated by adding distilled water and evaporation by replacing the pitcher fluid with a smaller volume of more concentrated pitcher fluid. For this experiment, we randomly selected 20 freshly opened pitchers from separate plants and measured their initial fluid volume by decanting the contents into a graduated Falcon tube. All pitchers were covered with a polypropylene sheet folded into a cone attached to the tendril of the pitcher to exclude rainwater ingress during the experimental period. Pitchers were randomly assigned to either of two treatments.

1. *Flooding.* To simulate the effect of rainfall, the fluid volume of ten pitchers was doubled by adding distilled water to the pitcher fluid.
2. *Evaporation.* To simulate the effect of hot, dry weather, the pitcher fluid of 10 pitchers was replaced with one-quarter of the original volume of a four times more concentrated pitcher fluid. This was produced by evaporating fresh pitcher fluid from other pitchers in a Petri dish under a fan for ∼12 h. The evaporation was terminated when the electrical conductivity of the fluid had quadrupled; fluid conductivity was measured with a portable conductivity meter (EC150, Extech Instruments, NH, USA) as a measure of ionic concentration.

For pitchers in both treatment groups, pitcher fluid level and the electrical conductivity of the fluid were recorded daily at 09.00 h over a period of 5 days for all 20 experimental pitchers. The conductivity–volume product was calculated as a proxy for the total number of ions in the pitcher by multiplying the conductivity and volume values.

### Experiment 4: age dependence of the ability of the pitcher to absorb and secrete fluid

To investigate whether the ability of the pitcher to regulate the fluid level changes with pitcher age, 20 freshly opened pitchers and 20 pitchers 4 weeks old (randomly selected from different plants and not used in any other experiment) were emptied and rinsed with distilled water. To investigate the ability of the pitchers to absorb and secrete fluid, pitchers were randomly assigned to one of two treatments.

1. *Replacement with distilled water.* Ten pitchers of each age group were filled with distilled water up to the original fluid level.
2. *Fluid removal.* The fluid of ten pitchers of each age group was removed, and the pitchers were left empty.

Pitcher fluid levels were recorded daily at 09.00 h for a period of 14 days for the distilled water treatment and 7 days for the fluid removal treatment, respectively. This was owing to the different time scales of the responses.

### Morphology of the inner pitcher wall

To investigate variation in the structure and size of the glands from the pitcher base to the upper end of the inner wall of *N. rafflesiana* pitchers, we imaged one freeze-dried sample of a pitcher wall, taking advantage of the ultraviolet (UV) autofluorescence of the glands. UV fluorescence microscopy was performed with a Leica DMR HC microscope, an HBO 103 W/2 mercury arc lamp and a UV filter system (excitation 340–380 nm, emission >425 nm) and recorded using a Nikon D750 camera mounted on the microscope. Extended focus images were obtained by merging *z*-stacks of images (20 µm intervals) in Adobe Photoshop. We imaged the pitcher wall at 20 mm intervals from the pitcher base to immediately below the peristome, in plan view. We measured the average open gland area using ImageJ2 (Rueden *et al.*, 2017) by manually tracing the outline of four glands at each sampling point.

### Statistical analyses

The fluid levels of 4-week-old vs. freshly opened pitchers and of pitchers with vs. without a lid were compared using independent *t*-tests (Exp. 1a, c). Daily changes in the fluid level of pitchers and control vials were compared using paired *t*-tests (Exp. 1b). The correlation of daily rainfall and daily fluid level changes was tested using a linear mixed-effects model, with rainfall and vessel (pitcher or vial) as fixed effects and with pitcher–vial pair number as a random effect (Exp. 1b). The effect of pitcher fluid level on prey capture rate was tested using a one-way ANOVA on four fluid level groups, followed by pairwise Tukey’s HSD tests (Exp. 2a). The effect of pitcher fluid level on prey capture efficiency was tested using pairwise 2 × 2 Fisher’s exact tests on the number of captured versus escaped prey at each fluid height, and Bonferroni–Holm correction (Exp. 2b). To compare the relative fluid volume, conductivity and conductivity–volume product for a set of pitchers before and after manipulations, we used paired *t*-tests (Exp. 3 and Exp. 4). For all data, normality was verified by inspecting diagnostic Q–Q plots. Statistical calculations were performed in Python (v.3.10.9).

## RESULTS

### Experiment 1: nnatural fluctuation of pitcher fluid level

*Experiment 1a: fluid level in freshly opened and 4-week-old pitchers.* Freshly opened *N. rafflesiana* pitchers were consistently about halfway filled with fluid. Their relative fluid level (*L*/*H*, see Fig. 2A) was independent of pitcher height (*t*_115_ = −1.246, *P* = 0.215) and amounted on average to 47.8 ± 7.3 % (mean ± s.d., range 31.3–75.6 %, Fig. 2B). We found a similar relative fluid level of 48.5 ± 6.2 % in a smaller set of 4-week-old pitchers; there was no significant difference in the average fluid level found in freshly opened pitchers (independent *t*-test, *t*_124_ = 0.3, *P* = 0.763).

**Fig. 3.**
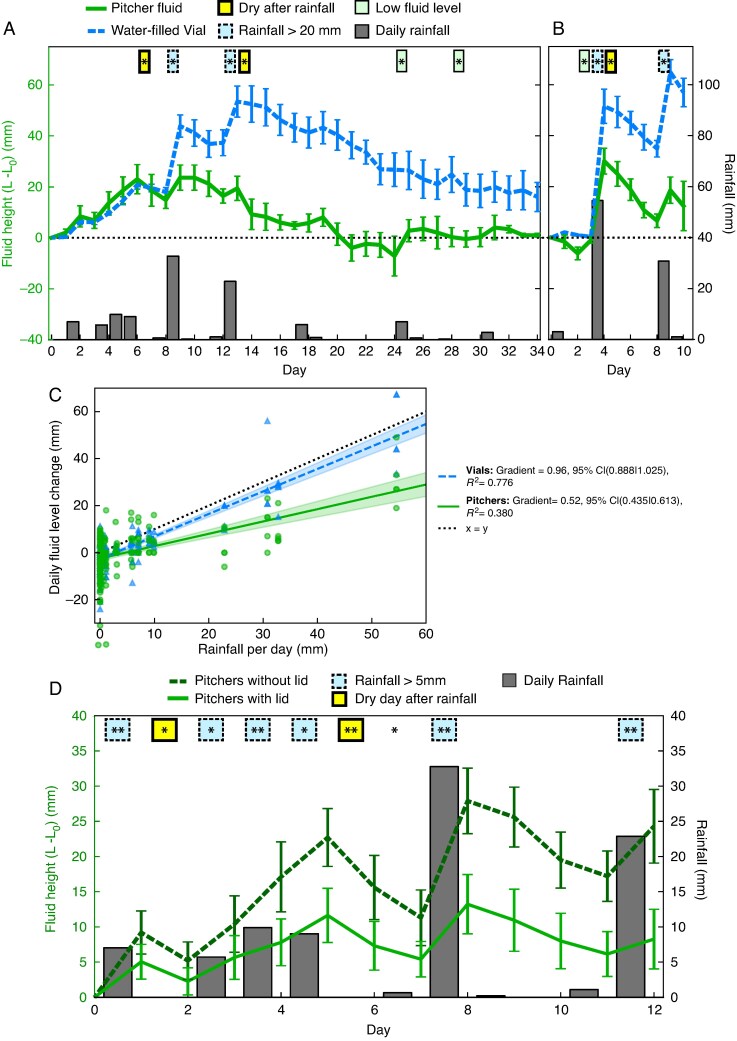
(A, B) Time series of the fluid level (relative to the initial level, *L*_0_) in *Nepenthes rafflesiana* pitchers (solid line, green) and water-filled control vials (dashed line, blue) measured every morning in field conditions: error bars represent the s.d. of daily measurements. Daily rainfall was measured from 09.00 to 09.00 h based on rain gauge data (grey bars). The differences between the daily fluid level changes of pitchers and vials were tested statistically on each day using paired *t*-tests, and all significant differences are marked (*P < 0.05, **P < 0.01 and ***P < 0.001); colours (line patterns) indicate characteristic conditions in which these significant differences occurred [yellow (dashed line), rain free after rainfall; blue (solid thick line), rainfall >20 mm; green (solid thin line), low/intermediate pitcher fluid level]. (A) Dataset A: five pitchers and adjacent control vials, monitored over 35 days. (B) Dataset B: five pitchers and adjacent control vials, monitored over 11 days. (C) Daily changes in fluid level for pitchers (green circles, solid line) and control vials (blue triangles, dashed line) from dataset A and B versus the daily rainfall. Linear regressions are plotted for each group, with the 95 % confidence intervals shaded. (D) Time course of the fluid level (relative to the initial level, *L*_0_) measured in pitchers with a lid (light green, solid line; *n* = 31) and with their lid removed (dark green, dashed line; *n* = 10). Fluid levels and rainfall were measured and plotted as in A and B, and differences in the daily fluid level changes between pitchers with and without lids were tested statistically on each day.

*Experiment 1b: daily changes of pitcher fluid level.* To monitor the changes in the pitcher fluid level over time, we measured the fluid level daily on two sets of five freshly opened *N. rafflesiana* pitchers and water-filled control vials attached adjacent to each pitcher. The pitcher fluid levels showed significant weather-related fluctuations, rising with rain and falling during dry periods (Fig. 3A, B). These fluctuations corresponded to relative fluid level ranging from 31.1 to 84.1 %. The magnitude of these fluctuations was significantly smaller in the pitchers than in the water-filled control vials (linear mixed-effects model on the absolute value of daily fluid level changes: *z* = 3.7, *P* < 0.001). When it rained, the fluid level of the pitchers increased less than in the control vials (linear mixed-effects model on daily fluid level changes; interaction rainfall × vessel: *z* = 15.0, *P* < 0.001). Although the increase in fluid level was approximately as high as the amount of rain fallen in the vials [ordinary least squares regression slope: 0.957, 95 % confidence interval (0.885; 1.028)], it was smaller in the pitchers [slope: 0.524, 95 % confidence interval (0.419; 0.629); Fig. 3C].

The daily fluid level changes in the pitchers differed in characteristic ways from the control vials; significant differences occurred for particular weather and/or pitcher conditions. Firstly, on all days of heavy rain (>20 mm, *n* = 4 days), significantly less water entered the pitchers (on average 33.9 ± 3.6 % of the rainfall) than the control vials (85.3 ± 9.9 % of the rainfall; see Supplementary Data Table S1). Secondly, on dry days (0 mm rain) following rainfall, pitchers lost fluid significantly faster than the control vials if their fluid level was >10 mm above the initial level (*n* = 3 days; see Supplementary Data Table S1). Thirdly, pitchers lost fluid significantly more slowly or even gained fluid on rain-free days if they were at or below their initial level; fluid level gains indicating fluid secretion were seen on two of these days (day 25, Fig. 3A; and day 3, Fig. 3B), and during sustained dry periods the pitchers maintained their fluid level and vials lost fluid (day 29, Fig. 3A; Supplementary Data Table S1). All these pitcher responses increased the fluid level in dry conditions or low fluid levels and reduced it in wet conditions or high fluid levels, thus achieving a homeostasis of the fluid level.

*Experiment 1c: effect of pitcher lid on pitcher fluid level.* Pitchers with and without their lid showed the same general pattern of fluid level increase and decrease in synchrony with naturally occurring rainfall (Fig. 3D). When it rained, however, the fluid level of the pitchers without a lid increased more than that of the intact pitchers (linear mixed-effects model on daily fluid level changes; interaction rainfall × lid: *z* = 6.2, *P* < 0.001). On all 6 days with >5 mm rain, significantly more water entered the pitchers without a lid in comparison to intact pitchers (Supplementary Data Table S2). As a result, the fluid levels of the lid-free pitchers after 13 days were 24.3 ± 2.6 mm (mean ± s.d.) above their starting level compared with 8.3 ± 2.1 mm in the intact pitchers [lid vs. no lid (final day), independent *t*-test, *t*_39_ = −3.9, *P* < 0.001].

### Experiment 2: influence of pitcher fluid level on prey capture rate and efficiency

**Fig. 4.**
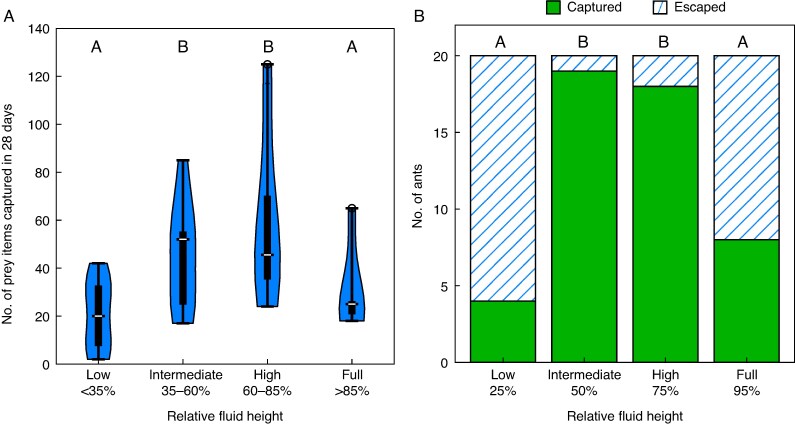
Dependence of prey capture rate and prey capture efficiency on relative pitcher fluid level. (A) Prey items naturally captured in 36 *Nepenthes rafflesiana* pitchers over a 28-day period during which relative fluid levels were maintained experimentally at low, intermediate, high or full levels in the field. (B) Number of *Polyrhachis triaena* ants escaped or captured by a cut-off pitcher, in which the relative fluid level was successively set to low, intermediate, high or full. Different letters indicate statistically significant differences between groups according to Tukey’s HSD *post hoc* test (A; see Supplementary Data Table S3) and Fisher’s exact pairwise comparisons with Bonferroni–Holm correction (B; see Supplementary Data Table S4); groups sharing the same letter are not significantly different.

*Experiment 2a: field measurement of prey capture rate.* The pitcher fluid level had a significant effect on natural prey capture (one-way ANOVA on square root-transformed data: F(3,32) = 5.8, *P* = 0.003, Fig. 4A). Pitchers with low fluid levels and full pitchers captured significantly less prey than the control. The difference between high and intermediate (control) fluid levels was not significant (Fig. 4A; Supplementary Data Table S3). There was no evidence that pitcher fluid level had a different effect on flying and non-flying prey (see Supplementary Data Fig. S1).

*Experiment 2b: laboratory measurements of prey capture efficiency.* When we observed and quantified the prey capture efficiency experimentally by measuring the trapping success for *P. triaena* ants that had fallen into the pitcher, the number of ants retained in the pitcher was also significantly higher for intermediate and high fluid levels than for low and full fluid levels (Fig. 4B; Supplementary Data Table S4). When the fluid level was low, we observed that many ants fell into the pitcher but landed on the dry pitcher walls and were then able to climb back to safety (see Supplementary Data Video 1). In contrast, when pitchers were nearly full, we observed that several ants hauled themselves horizontally onto the peristome and managed to escape (see Supplementary Data Video 2).

### Experiment 3: effect of experimental flooding and fluid evaporation on pitcher fluid levels

**Fig. 5.**
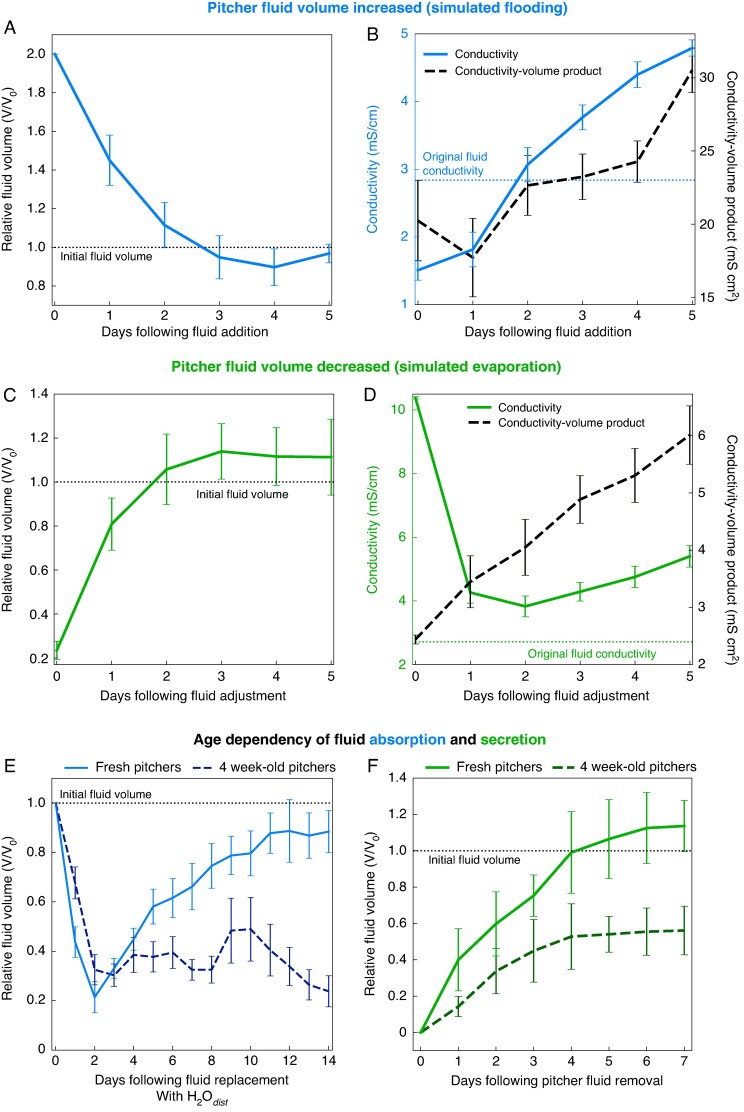
Effects of simulated rainfall (pitcher fluid increase; A, B) or dry weather (pitcher fluid evaporation; C, D) on the volume and conductivity of the fluid of freshly opened pitchers and the pitcher age dependence of these effects (E, F). (A, B) Fluid volumes were doubled experimentally by adding distilled water. (C, D) Fluid volumes were reduced experimentally by replacing the fluid with one-quarter of the original volume of a 4-fold concentrated pitcher fluid. (E) Pitcher fluid was replaced with distilled water up to the original level. (F) Pitchers were emptied and rinsed of their pitcher fluid. (A, C, E, F) Daily measurements of relative fluid volume (volume normalized by the initial volume for each pitcher). (B, D) Daily measurements of electrical conductivity (in millisiemens per centimetre) and corresponding product of conductivity and volume (a proxy for the total number of ions). (A–F) Lines show the means and error bars the s.d. of daily measurements (*n* = 10 per group).

When the fluid volume was doubled by adding distilled water, fluid volumes returned to a mean value of 96.8 ± 4.8 % (mean ± s.d.) of the original fluid volume within 3 days (Fig. 5A). This decrease was highly significant (day 0 vs. day 3, paired *t*-test, *t*_9_ = 33.5, *P* < 0.001). At the same time, the conductivity of the fluid increased to its original value within 2 days (day 0 vs. day 2, paired *t*-test, *t*_9_ = −5.4, *P* < 0.001) but then continued to increase (Fig. 5B) up to 170 % of the original value.

When the pitcher fluid was replaced by one-quarter of the volume of a 4-fold concentrated fluid, the fluid increased back to 105.7 ± 8.0 % (mean ± s.d.) of its pretreatment level within 2 days (day 0 vs. day 2, paired *t*-test, *t*_9_ = 5.0, *P* < 0.001), then stayed at this level (day 2 vs. day 5, paired *t*-test, *t*_9_ = −1.0, *P* > 0.05; Fig. 5C). The conductivity of the concentrated pitcher fluid decreased significantly over the course of the experiment (day 0 vs. day 5, paired *t*-test, *t*_9_ = 5.0, *P* < 0.001; Fig. 5D); it did not return fully to the original level of conductivity but decreased on day 2 to 36.9 ± 4.6 % (mean ± s.d.) of its value, then increased.

We calculated the product of conductivity and fluid volume as a proxy for the amount of ions (including protons) present in the pitcher. In both experiments, we observed a significant increase in this conductivity–volume product (day 0 vs. day 5, paired *t*-tests, added water: *t*_9_ = 3.3, *P* = 0.010; evaporated fluid: *t*_9_ = 6.0 *P* < 0.001), indicating that the pitchers secreted ions during both experiments. The pH of the pitcher fluid decreased during both experiments (Supplementary Data Fig. S2), suggesting that the secreted ions were mainly protons.

### Experiment 4: age dependence of the ability of the pitcher to absorb and secrete fluid

Both fresh and 4-week-old pitchers with their fluid replaced with distilled water showed a significant decrease in fluid volume compared with their initial volume over the first 2 days (Fig. 5E; day 0 vs. day 2, paired *t*-tests, fresh: *t*_9_ = 8.2, *P* < 0.001, 4 weeks old: *t*_9_ = 7.3, *P* < 0.001). This drop in absolute fluid level (average on day 1: 21.1 ± 8.6 mm in fresh pitchers, 15.2 ± 7.3 mm in 4-week-old pitchers) was much faster than the maximally observed decrease attributable to evaporation in water-filled vials (Fig. 3A, B; maximum decrease 7.5 mm day^−1^), providing clear evidence for fluid absorption by the pitchers. Following this initial rapid absorption, fresh pitchers replenished their fluid volume to their pretreatment level within 12 days (day 2 vs. day 14, paired *t*-test, *t*_9_ = 1.4, *P* > 0.05), indicating fluid secretion. In contrast, 4-week-old pitchers did not recover their fluid volume to the pretreatment level (day 2 vs. day 14, paired *t*-test, *t*_9_ = 7.0, *P* < 0.001).

Fresh pitchers that were emptied of their original pitcher fluid rapidly secreted fluid and replenished their pretreatment volume within 4 days (day 4 vs. original fluid level, paired *t*-test, *t*_9_ = 0.3, *P* > 0.05). Four-week-old pitchers subjected to the same treatment also secreted fluid, but were unable to restore their fluid volume fully and reached only about half of the pretreatment fluid volume over the course of the experimental period (day 4 vs. original fluid level, paired *t*-test, *t*_9_ = 4.9, *P* < 0.001; Fig. 5F).

### Morphology of the inner pitcher wall

**Fig. 6.**
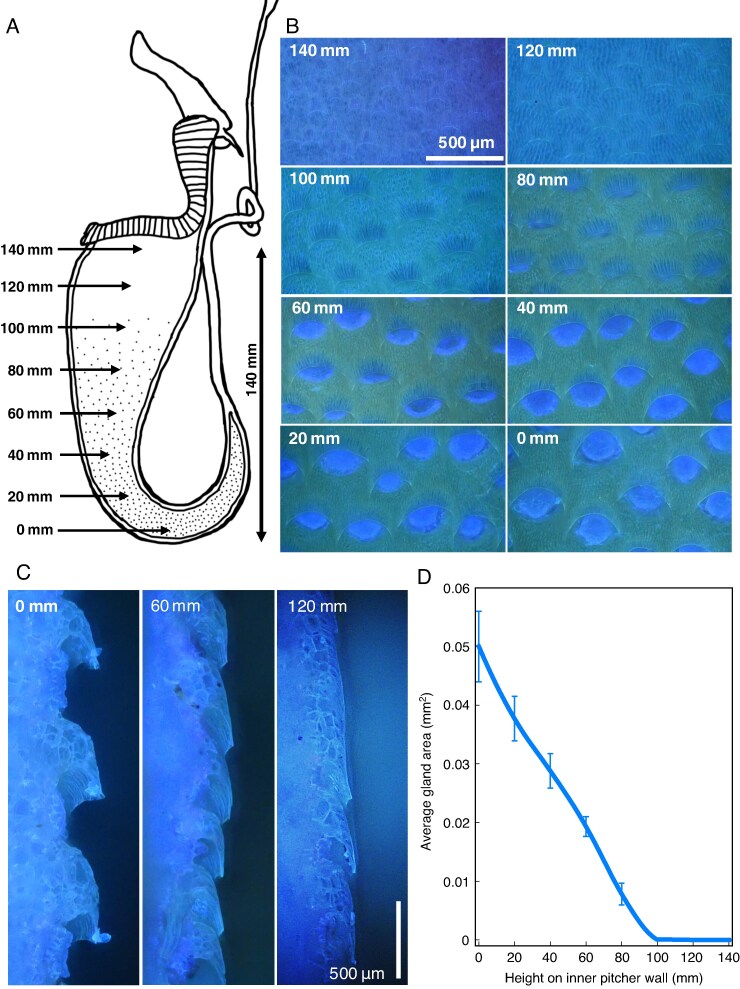
Morphology of the inner pitcher wall. (A) Diagram of a *Nepenthes rafflesiana* pitcher displaying the sampling points used in B–D. (B, C) Extended-focus ultraviolet-fluorescence images. (B) View of the pitcher wall from inside the pitcher, displaying the autofluorescent glands. Glands are covered to varying degrees by ‘hooded’ structures at different heights within the pitcher. (C) Longitudinal fracture of the inner pitcher wall, showing the varying profile of the hooded structures at different heights within the pitcher. (D) Average exposed gland area at different heights within the pitcher. Plot shows the mean areas of the four most central glands from the images in A; error bars represent the s.d.

The inner wall of upper *N. rafflesiana* pitchers showed a striking change in morphology from the bottom to the top of the pitcher (Fig. 6). The upper part of the pitcher wall lacked wax crystals and lunate cells, meaning that a clear separation between a ‘conductive zone’ and a ‘digestive zone’, as found in some other *Nepenthes* species (Lloyd, 1942), was missing. A regular pattern of multicellular glands covered the lower part of the pitcher wall at least up to 40 mm below the peristome, i.e. up to a level significantly above the fluid level in natural conditions (Fig. 6A). Each gland was embedded in an epidermal depression, which was partly covered by a ‘hood’ of epidermal cells overhanging the depression from its upper margin (Fig. 6B). The position of this hood relative to the gland changed from the bottom to the top of the pitcher. Although the glands were fully exposed at the bottom of the pitcher (average visible gland size 0.05 ± 0.006 mm^2^), they were increasingly covered by the hood further up on the pitcher wall and became invisible in plan view 40–60 mm below the peristome (Fig. 6B, D).

## DISCUSSION

The results of our study confirm our hypotheses about the fluctuation, ecological function and regulation of the fluid level in *N. rafflesiana* pitcher plants. In particular they show that:

1. Pitcher fluid levels fluctuate as a result of rainwater ingress and evaporation, despite the presence of a lid that partly shields the pitcher.
2. The fluid level strongly impacts the prey capture success of the pitcher, with most prey being captured at intermediate levels.
3. Pitchers actively maintain an intermediate fluid level by regulating it through the secretion and absorption of water.
4. The ability to regulate the fluid level decreases in older pitchers, because they can still absorb but no longer secrete fluid.
5. The glands on the inner pitcher wall of *N. rafflesiana* change in morphology from the bottom to the top of the pitcher, with possible implications for the mechanisms of fluid level control.

In the following, we will initially discuss the ecological consequences of pitcher fluid level, then the mechanisms underlying the homeostatic control of pitcher fluid volume.

### How does the pitcher fluid level affect prey capture?

The regulation of the pitcher fluid level is probably important for the dual function of the pitcher fluid in prey capture and digestion. Our findings demonstrate that *N. rafflesiana* pitchers with low and very high fluid levels caught significantly less prey. When fluid levels were low, insects falling into the pitchers (which taper towards the bottom) often did not land in the fluid but on the inner pitcher wall and were able to climb back out of the pitcher. Negative effects of low fluid levels on prey capture have also been reported from *Heliamphora* and *Sarracenia* pitcher plants (Jaffe *et al.*, 1992; Newell and Nastase, 1998). Trapping is also impaired in *N. rafflesiana* pitchers with very high fluid levels by the ability of insects to escape more easily over the rim of the pitcher. This effect has been hypothesized previously (Clarke, 1997) but not shown experimentally. Severe dilution by heavy rain could also affect the ability of the fluid to retain captured prey. For *N. rafflesiana*, Gaume and Forterre (2007) showed that the extensional viscosity of the fluid plays an important role in prey retention. Although this property was shown to be remarkably resistant to dilution, it ultimately decreased for strong dilution, with detrimental effects on prey retention. Moreover, Bazile *et al.* (2015) showed that pitcher fluid acidity was important for killing the prey. Given that acidic fluid is widespread in *Nepenthes* (Clarke, 1997), dilution of the pitcher fluid might reduce the ability of the trap to retain and kill prey in many *Nepenthes* species.

Pitcher fluid levels could rise quickly to high levels during heavy rain events, which would have a negative effect on prey capture. However, the rapid fluid absorption by the pitchers ensures that the rise in fluid level is only small, meaning that very high levels are not reached and there is no reduction in the capture rate. In contrast, drops in fluid level during dry spells are always relatively slow and can easily be compensated for by fluid secretion by the pitcher. This suggests that the ‘intermediate’ fluid level is favourable for *N. rafflesiana*, because the prey capture rate of pitchers suffers least from small fluid level increases owing to rainfall.

It is likely that the role of the fluid in digestion is also impacted by changes to volume and concentration. Prey is digested not only by plant-secreted enzymes (Amagase *et al.*, 1972; Matušíkova *et al.*, 2018), but also by a diverse assemblage of bacteria and predominantly arthropods that live in the pitcher fluid (Clarke and Kitching, 1993; Kitching, 2000; Bota *et al.*, 2025). This pitcher infauna can help to break up larger prey items to make them more accessible to the digestive enzymes of the plant and subsequent absorption (Lam *et al.*, 2017; Leong *et al.*, 2018). Although we did not make direct observations of infauna communities in this study, there is strong evidence that the volume and composition of fluid influence the infauna of carnivorous plants (Kingsolver, 1979; Adlassnig *et al.*, 2011; Gilbert *et al.*, 2020), and a complete drying out of the phytotelm would be fatal for most species. The control of the pitcher fluid level observed in this study prevents such extreme fluctuations in this microhabitat, creating more favourable and stable conditions. *Nepenthes* pitchers can alter not only the volume but also the composition and acidity of their fluid, allowing them to influence their bacterial and infauna communities strongly (Clarke and Kitching, 1993; Adlassnig *et al.*, 2011; Gilbert *et al.*, 2020). Thus, the ability of pitchers to regulate fluid level and composition is also likely to represent an advantage to digestion.

### What mechanisms contribute to the fluid level control in *N. rafflesiana* pitchers?

We found that fluid level and composition in *N. rafflesiana* pitchers is influenced by a combination of pitcher morphology and gland-mediated fluid transport. It has long been proposed that the lid in most species of *Nepenthes* and *Sarracenia* functions to exclude rainfall (Lloyd, 1942), and our findings confirm that the lid reduces the amount of rainfall into *N. rafflesiana* pitchers. Interestingly, a lid is not the only way to regulate fluid level in carnivorous plants. *Heliamphora* pitchers lack a lid; here, the flooding of the pitchers is prevented by a small drainage hole or slit, through which excess water can drain away (Jaffe *et al.*, 1992). The shielding by the lid is not complete in *N. rafflesiana*, because the fluid levels of unmanipulated pitchers also increased with each rainfall event. The incomplete shielding of the lid might be beneficial because it allows the peristome to be wetted by rain, which is essential for its slipperiness and role in insect capture (Bohn and Federle, 2004; Bauer *et al.*, 2008). The peristome is not only highly wettable but has also been shown to facilitate surface water transport from the inner to the outer margin (Chen *et al.*, 2016). Although not tested here, this directional water transport might also help to divert raindrops landing on the peristome towards the outside of the pitcher. Thus, both the lid and the peristome might contribute to fluid level regulation.

A further mechanical factor that could influence fluid level control is the shape of the pitchers. A study on the effect of pitcher shape on fluid level in *Sarracenia purpurea*, where fluid secretion was considered negligible, found that the elongated pitcher shape protects better against dehydration than a hypothetical hemispherical shape (Kingsolver, 1981). In contrast, a conical pitcher shape should lead to greater fluid level changes than a cylindrical shape owing to the larger rain catchment area relative to the overall volume.

In addition to the effects of pitcher lid and pitcher shape, the control of the pitcher fluid level is also determined by the secretion and absorption of fluid. Our findings show that *N. rafflesiana* pitchers are able both to secrete and to absorb fluid. Pitchers sometimes increased fluid levels on rain-free days, and emptied pitchers refilled, demonstrating their ability to secrete fluid. Secretion is also evident in *Nepenthes* pitchers prior to opening, because they are already fluid filled when they open (Buch *et al.*, 2013). Fluid secretion by immature pitchers has also been reported for Australian *Cephalotus* (Cephalotaceae) and American *Sarracenia* and *Darlingtonia* (Sarraceniaceae) pitcher plants (Jones and Hepburn, 1927; Lloyd, 1942; Clarke, 1988; Armitage, 2017). However, it has not previously been investigated whether mature, open pitchers maintain the ability to secrete fluid. The pitcher responses we observed to the experimental removal of the fluid or its replacement with more concentrated pitcher fluid clearly show that mature *N. rafflesiana* pitchers can rapidly secrete fluid to restore their original fluid level.

In contrast to fluid secretion, the ability of the pitcher to absorb fluid was apparent from the rapid drop in fluid level during our experimental simulation of flooding and the replacement of the pitcher fluid with distilled water. This ability was still fully present in 4-week-old pitchers.

Fluid secretion and absorption by leaves are not restricted to carnivorous plants but widespread. Water can not only be absorbed via the leaf surface (enhanced by pores and trichomes; Boanares *et al.*, 2018) but can also be excreted by nectaries, salt glands and leaf hydathodes, which are present in all vascular plants (Singh, 2014; Bellenot *et al.*, 2022).

The most likely sites of fluid secretion and absorption are the multicellular digestive glands on the inner surface of the pitchers, which have been reported to secrete and absorb ions, protons and digestive enzymes and to absorb nutrients (Lüttge, 1966; Owen Jr *et al.*, 1999; Schulze *et al.*, 1999; An *et al.*, 2001; Moran *et al.*, 2010). Investigations in *Nepenthes alata* showed that endodermis-like regions in the base of pitcher glands ensure that any transport of materials into or out of the glands must occur through the symplast (Owen Jr *et al.*, 1999; Schulze *et al.*, 1999). It is likely that the fluid transport by the glands is driven by ion pumping, similar to the water transport across the root endodermis. From the visualization of symplastic fluid transport with fluorescent tracers in *N. alata*, it was concluded that in unopened pitchers, the primary function of the glands is secretion, whereas in mature pitchers, secretion is blocked by an endodermal layer such that the glands primarily absorb fluid (Owen Jr *et al.*, 1999).

However, our results show that *N. rafflesiana* pitchers, and consequently their glands, retain the ability to secrete fluid even when mature (i.e. ≥4 weeks after opening). The ability of the pitchers to secrete fluid became weaker as they aged, and 4-week-old pitchers returned to only half the fluid level after fluid removal. This diminished fluid secretion might be related to the lower prey capture in older pitchers of *N. rafflesiana* (Bauer *et al.*, 2009); fluid level control might be less crucial in this phase, when pitchers primarily extract nutrients.

### What triggers the absorption or secretion of fluid by the pitcher glands?

To our knowledge, the control of fluid level and the underlying mechanisms have not been studied in any pitcher plant. The homeostatic control of fluid level via absorption and secretion in *N. rafflesiana* pitchers is likely to represent a negative feedback loop, in which the control variable is either the concentration of dissolved substances or the volume. If concentration is the control variable, absorption or secretion would be triggered by changes in ion concentration when the pitcher fluid becomes diluted by rain or concentrated by evaporation. Absorption or secretion of fluid could be passive and driven by the water potential gradient between gland cells and pitcher fluid or it could be coupled to active ion transport (Milburn, 1979). The rapid absorption of water that we observed when the pitcher fluid was replaced by an equal volume of distilled water clearly shows that absorption can be triggered by changes in concentration only. However, a concentration-based control mechanism alone (with a constant set-point concentration) could not explain the fluid level control we observed in *N. rafflesiana* pitchers. This is because not only the volume of the pitcher fluid but also the amount of solutes dissolved in it fluctuates as a result of prey capture and digestion and owing to ion secretion and absorption. We observed in our experiments that the ion concentration in the pitcher liquid increased over time, while the fluid level remained at an intermediate level. If the pitchers maintained only a constant concentration, we would expect to see pitchers with high or low fluid levels, depending on the amount of solute they contain.

Absorption or secretion could also be triggered by the changes in pitcher fluid level (volume) caused by rain or dry weather. One possible way in which such a volume-based control could be achieved is a different response to wetness by the gland cells in the upper and lower half of the pitcher. During flooding, gland cells in the normally dry upper part of the pitcher wall become wetted, and these cells could respond by absorbing water. When the pitcher fluid level drops below the usual range, the glands in the normally wet lower part of the pitcher wall become dry, and these cells could respond by secreting water. This hypothesized mechanism of volume control would require a physiological difference between the gland cells in the upper and lower part of the pitcher, as is suggested by the morphology of the glands (Moran *et al.*, 2010). In the population of *N. rafflesiana* we investigated, the pitchers mostly lack a clearly defined ‘wax crystal zone’ with lunate cells in the upper part of the pitcher wall, but the morphology of the lower and upper parts of the pitcher wall is nevertheless different. Consistent with observations of the structure of the glandular zone in other *Nepenthes* species (Adams and Smith, 1977; Gorb *et al.*, 2004), we found the exposed area of the glands to be much larger in the lower part of the pitcher, where they would typically be beneath the pitcher fluid surface (Fig. 6). In the upper part of the pitcher, the glands are partly or completely covered by ‘hoods’ formed by adjacent epidermal cells, which might also make it difficult for insect prey to latch on (Lloyd, 1942; Juniper *et al.*, 1989; Gorb *et al.*, 2004). A similar morphological transition from the bottom to the top of the pitcher has been reported for *N. bicalcarata* (Moran *et al.*, 2010). In *N. rafflesiana*, the most obvious change occurs approximately halfway up the pitcher, consistent with the target fluid level of the homeostatic control. This supports the hypothesis that volume control via locally different secretion or absorption at the inner pitcher wall plays an important role in controlling the fluid level.

Our results show that as pitchers age, their ability to secrete fluid is reduced. Given that the 4-week-old pitchers returned to only half the fluid level after fluid removal, it is likely that this ageing process proceeds from the top of the normally fluid-filled region to the bottom of the pitcher. This explanation is also consistent with previous reports that *N. rafflesiana* pitchers wither from the top to the bottom and that the lower part of the pitcher stays alive the longest (Bauer *et al.*, 2009).

Volume-based and concentration-based control are not mutually exclusive but could work together. Further research should focus on elucidating in more detail the control mechanisms in *N. rafflesiana* by investigating the contributions of pH control, ion absorption and ion secretion, in addition to the effects of evaporation and whole-plant water potential.

### Conclusion

Our study shows that the volume of fluid in the pitchers of *N. rafflesiana* plants is affected by rain and evaporation. The fluid level affects the prey capture success of pitchers, with both very low and very high fluid levels being unfavourable. Pitchers can actively maintain intermediate fluid levels by secreting and absorbing water, but the ability to secrete water decreases in older pitchers. This homeostatic fluid level control is a previously unrecognized adaptation of pitcher plants to their exposed habitats. Controlling fluid volume is probably essential for many other carnivorous plants that use open fluids for trapping and grow in open, exposed habitats. Understanding how carnivorous plants maintain their fluid-based traps is important, because many populations are threatened by habitat loss and increasingly extreme weather conditions caused by climate change.

## Supplementary Material

mcaf294_Supplementary_Data

## SUPPLEMENTARY DATA

Table S1: days with statistically significant differences between fluid level changes in pitchers and corresponding control vials (see Fig. 3A, B). Days are categorized as days with heavy rainfall (>20 mm, blue), dry days following rainfall (yellow) and days with low pitcher fluid level (green). Differences between pitchers and vials were tested statistically using paired *t*-tests. Table S2: days with statistically significant differences between fluid level changes in pitchers with and without lids (see Fig. 3D). Days are categorized as days with heavy rainfall (>10 mm, blue) and dry days following rainfall (yellow). Differences between pitchers with and without lids were tested statistically on all days using independent *t*-tests. Table S3: dependence of natural prey capture rate on relative pitcher fluid level (one-way ANOVA on square root-transformed data: *F*(3,32) = 5.8, *P* = 0.003, Fig. 4A); results of pairwise comparisons using Tukey’s HSD *post hoc* tests. Table S4: dependence of prey capture efficiency on relative pitcher fluid level (4 × 2 Fisher’s exact test, *P* < 0.001); results of pairwise 2 × 2 Fisher’s exact tests with Bonferroni–Holm correction. Figure S1: prey composition for different fluid heights of *N. rafflesiana* (same experiment as Fig. 4). Captured prey were sorted into flying and non-flying insects and the total number of each was counted. The data were then normalized by the number of prey items captured by each pitcher. The proportion of flying versus non-flying prey showed no significant dependence on the fluid level (Fisher’s exact test, *P* > 0.05). Figure S2: effect of experimental flooding (pitcher fluid increase) (A) and fluid evaporation (pitcher fluid evaporation) (B) on the fluid pH of freshly opened pitchers (same pitchers and conditions as in Fig. 5). (A, B) Lines show the means, and error bars the s.d. of daily measurements of pitcher fluid pH (*n* = 10 per group); black dotted line indicates the mean pH of the pitcher fluid before the experimental manipulation. Video 1: video showing a *Polyrhachis triaena* ant falling onto the dry walls of a *Nepenthes rafflesiana* pitcher and managing to escape when the fluid level is low. Video 2: video showing a *Polyrhachis triaena* ant escaping over the peristome of a *Nepenthes rafflesiana* pitcher when filled with fluid.
