# Supplementary material for: An insect trap adjusting to weather conditions: Nepenthes rafflesiana plants control the fluid level in their pitchers to maximize prey capture
Source: Ann Bot. 2025 Nov 12;137(3):833–46. doi: 10.1093/aob/mcaf294 (PMC12933680; doi:10.1093/aob/mcaf294)
Supplement: mcaf294_Supplementary_Data [file mcaf294_supplementary_data.zip › Supplementary_Material.pdf]

Supplementary Material

Table S1

Day (from data set A and B)	Rainfall in previous 24h (mm)	Fluid height change to previous day (mm)		Test statistic (df = 8)	p
		Pitchers	Vials		
**9A**	32.78	8.8	25.9	t=-4.2	0.003
**13A**	22.88	3.0	16.2	t=-3.3	0.011
**4B**	54.56	31.0	51.2	t=-2.4	0.044
**9B**	30.80	12.0	30.1	t=-2.3	0.046
**7A**	0.00	-4.4	-1.3	t=-2.6	0.033
**14A**	0.00	-10.2	-1.0	t=-2.4	0.041
**5B**	0.00	-5.0	-2.2	t=-2.7	0.026
**25A**	0.00	6.7	-0.2	t=3.6	0.007
**29A**	0.22	-0.6	-5.6	t=2.9	0.019
**3B**	0.00	5.2	-0.6	t=3.1	0.015

Table S2

Day	Rainfall in previous 24h (mm)	Pitcher fluid height change to previous day (mm)		Test statistic (df = 39)	p
		Pitchers with lid	Pitchers without lid		
**1**	7.04	5.0	9.2	t=-2.9	0.014
**3**	5.72	3.4	5.2	t=-2.1	0.036
**4**	9.90	2.1	6.7	t=-5.4	0.000
**5**	9.02	3.8	5.6	t=2.2	0.033
**8**	32.78	7.8	16.6	t=-4.9	0.000
**12**	22.88	2.1	7.1	t=-3.5	0.003
**2**	0.00	-2.8	-4.0	t=2.2	0.036
**6**	0.00	-3.0	-6.1	t=4.2	0.000

Table S3

**Comparison**	**diff**	**Lower**	**Upper**	**p_{adj}**
Low - Intermediate	-2.3	-4.5	-0.1	0.033
Low - High	-3.3	-5.6	-1.1	0.002
Low - Full	-1.2	-4.0	1.5	0.627
Intermediate - High	-1.0	-3.2	1.2	0.635
Intermediate - Full	2.2	-1.6	3.8	0.034
High - Full	2.1	-0.7	4.8	0.019

Table S4

**Comparison**	$\mathbf{p_{adj}}$
Low - Intermediate	<0.001
Low - High	0.001
Low - Full	1.000
Intermediate - High	1.000
Intermediate - Full	0.022
High - Full	0.040

Figure S1

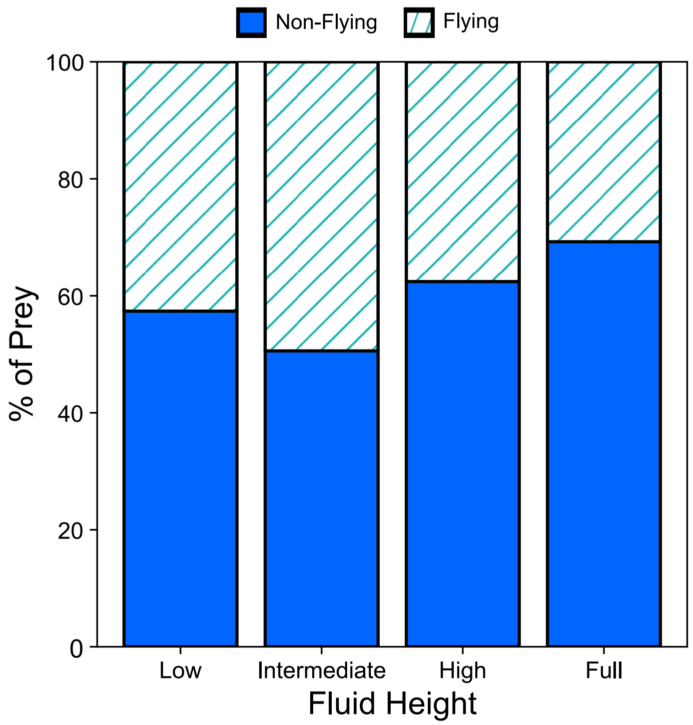

Figure S2

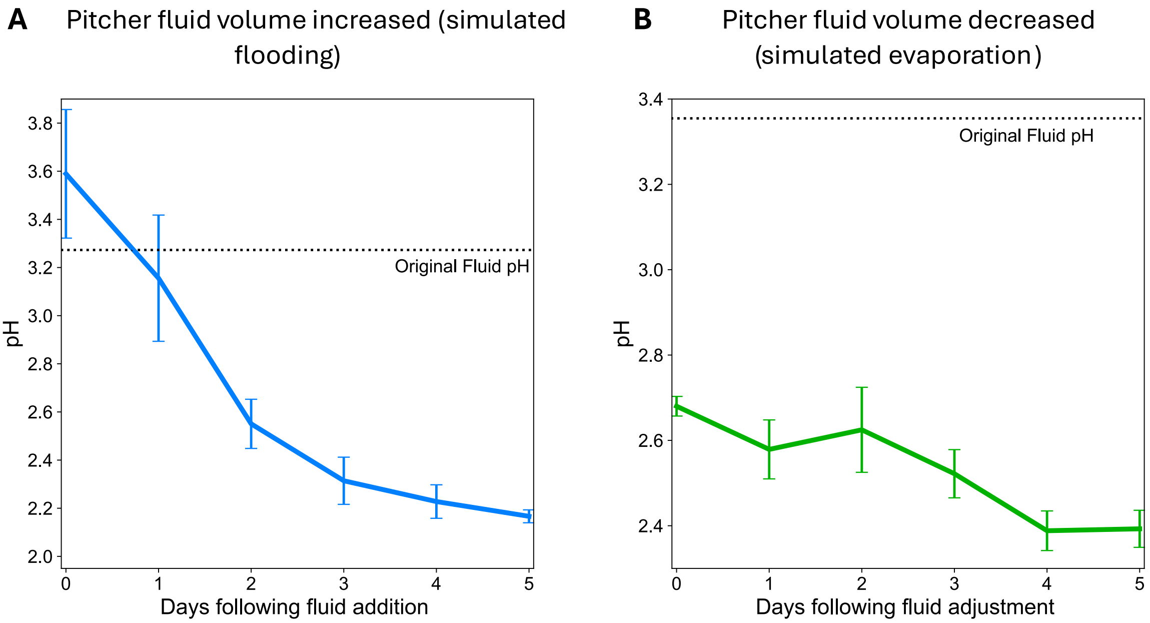

Video 1 – attached separately

Video 2 – attached separately

Table S1- Days with statistically significant differences between fluid level changes in pitchers and corresponding control vials (see Fig. 3A, B). Days are categorised as days with heavy rainfall (>20mm, blue), dry days following rainfall (yellow) and days with low pitcher fluid level (green). Differences between pitchers and vials were tested statistically using paired t-tests.

Table S2- Days with statistically significant differences between fluid level changes in pitchers with and without lids (see Fig. 3D). Days are categorised as days with heavy rainfall (>10mm, blue) and dry days following rainfall (yellow). Differences between pitchers with and without lids were tested statistically on all days using independent t-tests.

Table S3- Dependence of natural prey capture rate on relative pitcher fluid level (one-way ANOVA on square root transformed data: $F_{3,32}$= 5.8, p = 0.003, Fig. 4A); results of pairwise comparisons using Tukey's HSD Post-hoc tests.

Table S4- Dependence of prey capture efficiency on relative pitcher fluid level (4x2 Fisher exact test, p<0.001); results of pairwise 2x2 Fisher Exact tests with Bonferroni-Holm correction.

Figure S1- Prey composition for different fluid heights of *N. rafflesiana* (same experiment as Fig. 4). Captured prey were sorted into flying and non-flying insects and the total number of each was counted. The data was then normalised by the number of prey items captured by each pitcher. The proportion of flying vs. non-flying prey showed no significant dependence on the fluid level (Fisher Exact test, $p>0.05$).

Figure S2- Effect of experimental flooding (pitcher fluid increase) (A) and fluid evaporation (pitcher fluid evaporation) (B) on the fluid pH of freshly opened pitchers (same pitchers and conditions as in Fig. 5). (A,B) Lines show the means, and error bars the standard deviations of daily measurements of pitcher fluid pH ($n=10$ per group); black dotted line indicates the mean pH of the pitcher fluid before the experimental manipulation.

Video 1- Video showing a *Polyrhachis triaena* ant falling onto the dry walls of a *Nepenthes rafflesiana* pitcher and managing to escape when the fluid level is low.

Video 2- Video showing a *Polyrhachis triaena* ant escaping over the peristome of a *Nepenthes rafflesiana* pitcher when filled with fluid.
